# The Variety of Mechanosensitive Ion Channels in Retinal Neurons

**DOI:** 10.3390/ijms25094877

**Published:** 2024-04-30

**Authors:** Ji-Jie Pang

**Affiliations:** Department of Ophthalmology, Baylor College of Medicine, Houston, TX 77030, USA; jpang@bcm.edu

**Keywords:** mechanical-sensitive ion channels, retina, photoreceptor, bipolar cell, amacrine cell, horizontal cell, ganglion cell, BK, TRAAK, TREK, TRPV, ENaC, Piezo, TRP

## Abstract

Alterations in intraocular and external pressure critically involve the pathogenesis of glaucoma, traumatic retinal injury (TRI), and other retinal disorders, and retinal neurons have been reported to express multiple mechanical-sensitive channels (MSCs) in recent decades. However, the role of MSCs in visual functions and pressure-related retinal conditions has been unclear. This review will focus on the variety and functional significance of the MSCs permeable to K^+^, Na^+^, and Ca^2+^, primarily including the big potassium channel (BK); the two-pore domain potassium channels TRAAK and TREK; Piezo; the epithelial sodium channel (ENaC); and the transient receptor potential channels vanilloid TRPV1, TRPV2, and TRPV4 in retinal photoreceptors, bipolar cells, horizontal cells, amacrine cells, and ganglion cells. Most MSCs do not directly mediate visual signals in vertebrate retinas. On the other hand, some studies have shown that MSCs can open in physiological conditions and regulate the activities of retinal neurons. While these data reasonably predict the crossing of visual and mechanical signals, how retinal light pathways deal with endogenous and exogenous mechanical stimulation is uncertain.

## 1. Introduction

Pressure stresses are associated with a wide spectrum of retinal dysfunctions. Glaucoma is a blinding disease characterized by elevated intraocular pressure (IOP) and histological damage to the optic nerve, optic disk, and retinal ganglion cells (RGCs). It is the second leading cause of irreversible blindness in the world, and the worldwide prevalence of glaucoma in the population above 40 years is ~3.5% [1,2]. Glaucoma patients tend to develop “tunnel vision” over years if left untreated, a loss of the peripheral and paracentral visual fields. The higher vulnerability of RGCs in the peripheral vision is generally similar between glaucoma and pressurized air wave-caused traumatic retinal injury (TRI) [2,3,4,5]. Glaucoma pathogenesis is variably associated with age, race, genetic defects, glutamate excitotoxicity, and the elevation of IOP. The mean IOP level and the fast and slow fluctuations have been identified as the most significant risk factor of glaucoma [3,6,7]. Moreover, changes in the external pressure during flight, diving, etc. can also affect visual functions [8].

The peripheral vision is dominated by rod pathways. In mammals, cone photoreceptors have a lower light sensitivity and a distribution peak at the central retina/fovea (except for the S-cone), which provide the best visual resolution of the central retina in the daytime. Rods, on the other hand, dominantly occupy vast regions of the peripheral and paracentral retina, offering higher light sensitivity. Rod signals go through three pathways to reach RGCs: (1) the primary rod pathway formed by rods → rod bipolar cells (RBCs) → AII amacrine cells (AII ACs)—cone-driving depolarizing BCs (cDBCs) → RGCs, (2) the secondary rod pathway formed by rods—cones → cBCs → RGCs, and (3) the tertiary rod pathway composed by rods → hyperpolarizing BCs (HBCs) → RGCs, where “→” indicates glutamatergic synapses and “—” indicates electric synapses. The central vision is dominated by cones. Cone signals may reach RGCs via cones → cDBCs → ON and ON–OFF RGCs and cones → cHBCs → OFF and ON–OFF RGCs. It is uncertain whether mechanical signals can differentially affect retinal neurons in the rod and cone pathways.

**Figure 1:**
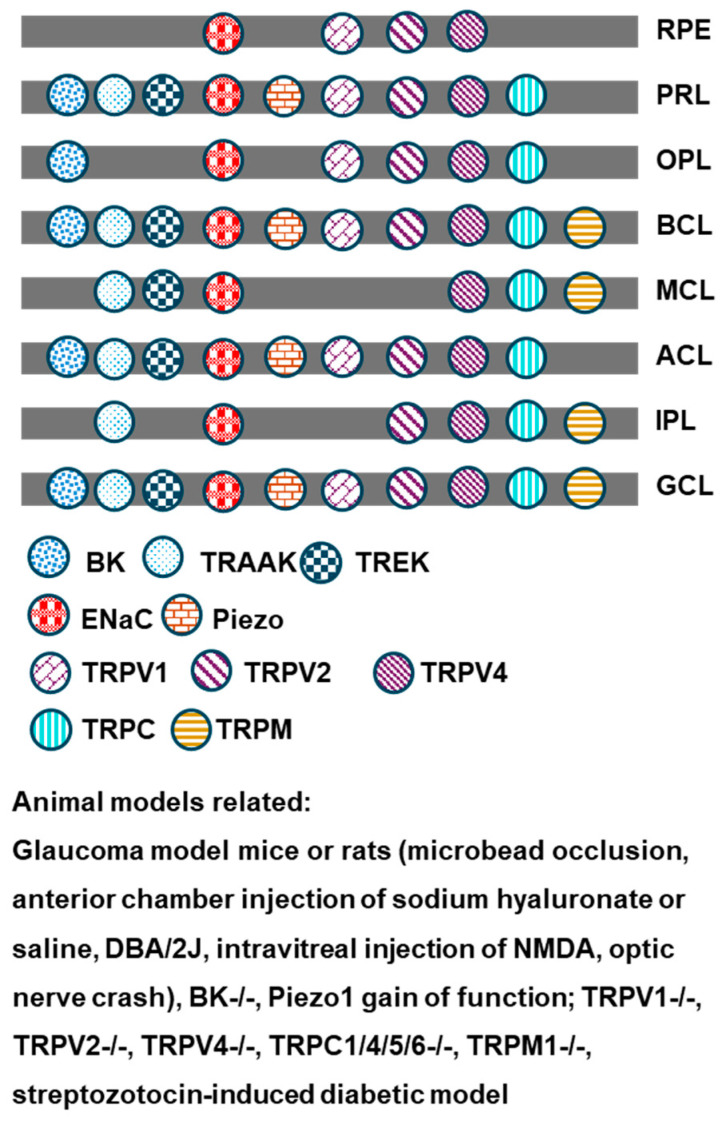
The distribution of MSCs in retinal layers. RPE—retinal pigment epithelium; PRL—photoreceptor layer; OPL—outer plexiform layer; BCL—bipolar cell layer; MCL—Müller cell layer; ACL—amacrine cell layer; IPL—inner plexiform layer; GCL—ganglion cell layer.

All eukaryotes express mechanical-sensitive ion channels (MSCs) to deal with a wide variety of physical stimuli [9]. Multiple types of MSCs have been reported in retinal neurons, such as the big potassium channel (BK); the two-pore domain potassium channels (K2Ps) TRAAK and TREK; Piezo; the epithelial sodium channel (ENaC); and the transient receptor potential channels vanilloid TRPV1, TRPV2, and TRPV4. These channels have shown variable distribution patterns among different types of retinal neurons (Table 1 and Figure 1), implying that the neurons employ different strategies and generate variable responses to mechanical stresses. These MSCs have been demonstrated as mechano-gated channels in other sensory neurons, Xenopus oocytes, or cultured cell lines, as they open directly by membrane tension and transduce the mechanical signals into electrical currents [10,11] (Table 2). MSCs may be activated by pressure stresses, changes in osmolarity and temperature, physical deformation, pH, and other modulators. These physical factors are often present in physiological conditions, and some channels are known to be constitutionally open (see below). The high IOP of glaucoma can significantly enlarge eyeballs in children, known as buphthalmos, and it also focally expands the retina at the cupping region of the optic disk in chronic glaucoma patients. Moreover, aging may directly induce structural remodeling and retinal deformation [12]. Hence, MSCs in retinal neurons may be activated in physiological and pathological conditions.

The presence of MSCs in neurons brings up the possibility that cellular mechanical homeostasis can critically determine the functional state and noise level of neuronal signals. This article will briefly review the literature on several representative MSCs in retinal photoreceptors, BCs, horizontal cells (HCs), ACs, and RGCs, providing a preliminary assessment of the significance of MSCs in light pathways. Per the journal’s restriction on citing more references of early publishing dates, many valuable reports were unable to be included for a better focus on the data from recent years.

Researchers identified MSCs in the retina by the gene or protein expression with PCR, Northern blot, Western blot, immunocytochemistry, situ hybridization, etc. Immunocytochemistry, situ hybridization, and flow cytometry allow the analysis of MSCs in different neuronal subtypes. The channels’ activity in individual neurons is often studied with patch-clamp techniques and pharmacological reagents at the cellular and single-channel levels. Calcium imaging may measure calcium influxes via calcium permeable MSCs, such as TRPs and Piezo channels, but not others. Electroretinography (ERG) provides a convenient and noninvasive approach for functional assessment optimal for use with human patients, and it primarily examines the function of outer retinal neurons. ERG records the averaged field potential primarily from rods, cones, and ON BCs with small signals from Müller cells and third-order neurons, and due to the short duration of light flash often used in ERG recording, ~5 ms for animals and 0.1–0.2 s for patients [42,43], OFF responses are usually not evoked or evaluated. Since rods are nearly 10^3^ more sensitive than cones [44,45], the scotopic ERG is useful for evaluating the function of rods and highly light-sensitive rod-driven ON BCs (DBC_R1_) and rod-cone-mixed ON BCs (DBC_R2_, DBC_R/C_) [45], and the photopic ERG may assess the function of cones and cone-driven ON BCs. So far, researchers have created multiple transgenic animal models available for dissection of the role of MSCs, and quite a few have been applied to retinal and ophthalmological studies.

## 2. Properties of MSCs and the MSCs Expressed in Photoreceptors

### 2.1. MSCs Permeable to K^+^

The BK channel is also known as the calcium- and voltage-gated large conductance potassium channel, Maxi-K, KCNMA1, Slo1, Kca1.1, and stretch-activated potassium channels. It is a homotetrameric channel of four identical, pore-forming, and Ca^2+^ and voltage-sensing units with or without associating with the regulatory β and γ subunits. The single channel conductance is between 200 and 300 pS (reviewed by [13].)

BKs were reported around two decades ago in rods in goldfish and salamander retinas associated with membrane hyperpolarization and glutamate release [46,47]. Cones from rabbit and salamander retinas also express BK [15,48]. In line with previous data [46,47], BK blocker iberiotoxin increases the light response of some rods [15]. Studies from other tissues have revealed the mechanical sensitivity of BKs [49,50].

TREK-1 (KCNK2) and TRAAK (KCNK4) are the two-pore (2P) domain K^+^ channels (K2Ps) opened by membrane stretch as well as arachidonic acid (AA). TRAAK and TREK give rise to leak (also called background) K^+^ currents. A well-known role of background K^+^ currents is to stabilize the negative resting membrane potential and counterbalance depolarization. TRAAK was recently reported to open upon ultrasound, and the single channel conductance was ~73 pS [17]. Without the stimulation, TRAAK had an average open probability of 1.9%, while ultrasound and pressure increased the channel opening probability to 6.3% and 26%, respectively.

TRAAK has been reported in mouse [14,51] and salamander photoreceptors [15]. Mouse photoreceptors also express TREK1 [52]. Our team applied pressure on the rod inner segment (IS) and evoked sustained currents of three components [15]. The pressure-induced outward current at membrane potentials ≥−80 mV (*I_po_*) was sensitive to intracellular Cs^+^ and ruthenium red, in line with the expression of MSCs permeable to K^+^.

Fink and colleagues cloned and expressed TRAAK in Xenopus oocytes and COS cells and first reported the instantaneous and non-inactivating currents of TRAAK. The currents were not gated by voltage, only partially inhibited by Ba^2+^ at high concentrations, and were insensitive to the other classical K^+^ channel blockers tetraethylammonium (TEA), 4-aminopyridine, and Cs^+^. TRAAK could be stimulated by the neuroprotective drug riluzole [14,51], aprepitant [53], arachidonic acid (AA), and other unsaturated fatty acids but not by saturated fatty acids [14]. Wang and colleagues [51] explored the expression of TRAAK and the apoptosis of the outer nuclear layer (ONL) with immunostaining, Western blotting, and real-time polymerase chain reaction (RT-PCR). The channel agonist riluzole activated TRAAK and delayed the apoptosis of photoreceptors in the ONL of rd1 mice with retinal degeneration (Pde6brd1). They pointed out that the activation of TRAAK in rd1 mice protects photoreceptors from apoptosis.

TRAAK, TREK1, and BK are permeable to K^+^, and a typical reversal potential (Erev) for K currents (E_K_) is around −75 to −90 mV [17,31] (Table 2). TREK is sensitive to the blockage of Cs^+^ [54]. Using TEA to block most potassium channels largely depolarized salamander rods (33 mV, 82.5%), and TEA reduced the light response by 24% [15], suggesting the potential impact of K-permeable MSCs.

### 2.2. MSCs Permeable to Na^+^ and Ca^2+^

The retina expresses multiple types of TRPs; TRPs are involved in various medical conditions (reviewed by [55,56]), but not all of them are known as mechano-gated channels. TRPs include seven subfamilies, namely TRPC (canonical), TRPV, TRPM (melastatin), TRPN (NOMPC), TRPA (ANKTM1), TRPP (polycystin), and TRPML (mucolipin) [57,58]. TRPs share the common feature of six transmembrane domains and various degrees of sequence similarity and permeability to cations. TRPs are variably modulated by membrane tension, osmolality, temperature, phorbol esters, and G-protein-mediated regulation. TRPA1, TRPC1/3/5/6; TRPM3/4/7, TRPP1/2, TRPV2, and TRPV4 are activated by stretch, osmolality, or/and pressure and identified as mechano-gated channels (reviewed by [22,40]) (Table 2).

TRPs have been found in all retinal layers [59] (Table 1). Photoreceptors, ONL, and OPL express TRPV1, TRPV2, TRPV4, TRPM7, TRPP2, TRPC1, and TRPML1 in the vertebrate and mammalian retina [15,59,60]. There are still disputes on whether the outer retinal neurons express TRPV4. In TRPV4 knock-out mice, Yarishkin and colleagues [61] did not find significant changes in ERG a-waves and b-waves evoked by whole-field lights of 0.00025–79 cd.s/m^2^, and they concluded that TRPV4 did not regulate the distal retinal light responses. Several studies observed TRPV4 in retinal Müller cells, microglia cells, or astrocytes to critically involve volume regulation and swollen-related pathologies [62,63,64], which indicates the importance of TRPV4 in glial cells. On the other hand, TRPV4 signals show a horizontal distribution pattern in the OPL in the mice, porcine, primate, and salamander retinas [15,31,59,65], which are not very consistent with the vertical orientation of Müller cells and dispersed distribution of microglial cells. In mice with acute retinal detachment, the number of apoptotic photoreceptors was reduced by approximately 50% in TRPV4 knockout mice relative to wild-type mice [66].

TRPVs include TRPV1–6, in which TRPV1, TRPV2, and TRPV4 are sensitive to membrane stretch, pressure, fluid low, and/or osmotic pressure. TRPV4 opens by pressure [30], membrane stretch [67], warm temperature, and specific pharmacological agonists like GSK1016790A (GSK101, 1–2 μM) and 4α-phorbol 12,13-didecanoate (4αPDD, 1–2 μM) [58,68]. Piperlongumine was recently identified as a selective, reversible, and allosteric antagonist of human TRPV2 (hTRPV2) [69].

TRPVs carry cation currents that reverse at ~−10 to 10 mV [15,28,31,32]. The channel opening mediates membrane depolarization and neuronal excitotoxicity [70,71]. Our recent data revealed the expression of TRPV4 and TRPV2 in the axon terminal of rods and cones of the salamander retina [15]. Applying a positive pressure onto the inner segment (IS) of salamander rods elicited three components of currents, and one of them is a cation current that reverses at ~−10 mV. Meanwhile, hypotonicity induces a slow cation current of a similar reversal potential [15]. The data align with the immunocytochemical data for the expression of TRPVs. Also, pressure applied to the outer segment (OS) of rods and cones closed a Ca^2+^-dependent cation conductance reversed at ~0 mV, in line with the closing of TRPV2 in the OS [15]. The wild-type rat TRPV2 has been reported to be constitutively fully open [72], and the spatial structure of the agonist-free full-length TRPV2 molecular [73] showed larger upper and lower gates than the agonist-opened TRPV1. The pressure response in photoreceptors saturates at 25.9 mmHg. The agonists and antagonists of TRPV2 and TRPV4 variably affect the light response of salamander rods [15]. The light-evoked potentials recorded at various light intensities were larger at 23 °C than at 31 °C and severely disrupted at 43 °C. The results indicate that mechanical stimuli may affect light signals in outer retinal neurons via TRPVs.

ENaC belongs to the ENaC/degenerin (ENaC/DEG) family and involves the functions of sensing and responding to mechanical and chemical stimuli. It is highly Na^+^- and amiloride-sensitive (EC_50_ 150 nM). The permeability (P) ratio of PNa:PK = 100:1. ENaC/DEG is characterized by a relatively long extracellular loop bounded by two transmembrane pore-forming helices (TM1 and TM2). The single-channel conduction is 7.4 pS in low-Na^+^ solution and 7.5 pS in high-Na^+^ solution (reviewed by [26]).

ENaC is a heteromultimeric channel usually composed of three homologous subunits (α, β, and γ) with a 30% to 40% identity at the level of their amino acid sequence [26,74]. The immunoreactivity and gene expression of the pore-forming ENaC α-subunit have been observed in mammalian photoreceptors. The specific blocker amiloride [75] enhanced the ERG a-wave in the rat and rabbit retinas. Unlike other family members, ENaC is constitutively active [26,76], which, in line with the observation in photoreceptors [75], suggests that ENaCα may regulate visual signals in photoreceptors in physiological conditions. AP 301 is an agonist of ENaCα that has been trialed for treating pulmonary permeability edema [77].

About one decade ago, Coste and colleagues revealed a novel family of mechanically activated cation channels in eukaryotes, consisting of Piezo1 and Piezo2 channels. Piezo is the nonselective cationic mechanosensitive channel that is permeable to alkali ions (K^+^, Na^+^, and Cs^+^), divalent cations (Ba^2+^, Ca^2+^, Mg^2+^, and Mn^2+^), and several organic cations (tetramethyl ammonium (TMA), TEA, etc.). Like TRPVs, ENaC and Piezo carry cation currents, which reverse at ~0 mV [10,78] (Table 2). Hence, the channel opening is anticipated to mediate membrane depolarization and excitotoxicity.

Piezo1 mRNA was detected in the mouse ONL [79]. Immunocytochemical and electrophysiological data from Bocchero and colleagues identified Piezo 1 and TRPC1 in the rods of Xenopus retinas [80]. Mechanical stimulation in the order of 10 pN applied to OS or IS evoked “calcium transient”, and the channel blocker reduced the duration of the photo response to bright flashes. Bright flashes of light also caused a rapid shorting of OSs [80]. The authors proposed that MSCs, including TRPC1 and Piezo, play an integral role in rod phototransduction in the vertebrate retina. Light-induced shortening of OSs of rods and cones has been observed in humans and other vertebrates [80,81,82]. The change in the membrane tension/displacement occurred in milliseconds and was found to be positively correlated with the change in membrane potentials in cones like in other neurons [81]. The membrane deformation can theoretically activate MSCs in OSs, and it is unclear whether the latter may also trigger the former.

To sum up, MSCs may mediate both depolarizing and hyperpolarizing currents, and photoreceptors in vertebrate and mammalian retinas possess BK, TRAAK, TREK1, ENaCα, Piezo1, TRPV1, TRPV2, TRPV4, and TRPC1/4, putting the activities of photoreceptors under the influence of mechanical stresses.

## 3. MSCs in HCs

It has been uncertain how MSCs are expressed in retinal interneurons, including HCs, BCs, and ACs. Some earlier works revealed insignificant loss of neurons in the inner nuclear layer (INL) in glaucoma and mild pressure-caused retinopathy in the outer retina. Somas of BCs, ACs, and Müller cells reside in INL; BCs in the upper half of the INL; ACs in the lower half; and Müller cells in the middle [83]. The inner plexiform layer (IPL) is composed of the axon terminals of BCs, processes of ACs, and dendrites of RGCs, and the retinal outer plexiform layer (OPL) consists of the axon terminals of photoreceptors, dendrites of BCs, and processes of HCs.

In dissociated HCs from the retinas of the rat and mouse [84], Sun and colleagues first identified a BK-mediated outward current. The single-channel conductance measured in symmetrical 150 mM K^+^ in mouse HCs was ~250 pS (202–279 pS). The BK-mediated membrane current was identified by the blockage of BK antagonists iberiotoxin (100 nM) and paxillin (2.5 μM), as well as Ca^2+^-free solutions, divalent cation, and voltage-gated calcium channel blockers. The potassium current was outwardly rectified and reversed around −75 mV. Blocking BK with paxillin depolarized the membrane and produced oscillation of increasing frequency, and the synthetic BK agonist NS1619 inhibited these oscillations. The authors concluded that the activation of BKα channels put a ceiling on membrane depolarization and regulated the temporal responsivity of HCs. This finding is consistent with the notion that BK hyperpolarizes HCs, counterbalancing the membrane depolarization and reducing excitatory visual noises. In salamander retinas [15], the TRPV2 antibody brightly labeled the OPL, and half of the calretinin/GABA-positive processes of horizontal cells (HCs) were labeled for TRPV2. Pressure applied on the IS of salamander rods could evoke a Co^2+^-sensitive inward current component in rods at membrane potentials < −50 mV (*I_pi_*). The data indicate the presence of TRPs in a presynaptic site, likely the processes of HCs.

Hence, HCs express BK and TRPV2, which can modulate the membrane potential and synaptic outputs of HCs. It has been unclear whether other MSCs in the OPL belong to HC processes.

## 4. MSCs in BCs

BK was first observed in isolated ON BCs from the goldfish retina [85,86] to mediate resonance (60–70 Hz or 5–10 Hz) K^+^ currents sensitive to 100 nM charybdotoxin [85]. The BK-mediated potassium current was coupled with L-type voltage-gated calcium channels (L-CaV). Nagai and colleagues used in situ hybridization and immunohistochemistry and observed BK in rabbit RBCs and cone ON BCs [48]. In BKα mutant mice, the ERG b-wave at the mesopic range (high scotopic range) was reduced, while others were not affected, indicating that BK involves the function of ON BCs [87,88].

BCs express TRAAK [14] and BK [87] in mouse and rat retinas. The somas of BCs reside in the INL, where TREK1, TREK2 [52], and TRAAK [14,52] have also been observed in mouse retina. Gao and colleagues observed TRPV4 in the dendrites and somas of BCs in primate retina [31]. In individually recorded BCs, long and short pressure steps both evoked transient cation currents, which reversed at ~−10 mV and were enhanced upon heating from 24 °C to 34 °C, in line with TRPV4. The pressure for the half-maximal effect in the primate retina was ~20 mmHg [31] comparable to the borderline of harmful IOP level in glaucoma. The transient responses indicate that the channels in BCs may adapt to sustained mechanical stimuli but respond to pressure changes, further suggesting that BCs may respond to the fluctuation in IOP levels under physiological and pathological conditions.

The mRNA and immunoreactivity of ENaCα have been observed in BCs in mammalian retina. The function of BCs is accessible in vivo with ERG. In the presence of ENaC blocker amiloride, Brockway and colleagues [75] found that the ERG a-, b- and d-waves were all enhanced in the rat retina, while the slow PIII-Müller cell response [75] was reduced. The data indicate that higher activities of ENaC can reduce the function of ON BCs in normal conditions. Moreover, Piezo1 mRNA was detected in the mouse INL [79].

The blast-induced pressure wave can reduce the ERG b-wave at post-blast 3–7 days [89,90] and damage the dendrites of RBCs [91]. High IOP also reduces BC synapses in RGC dendrites [92,93,94,95]. However, in TRI studies, the data on ERG b-wave observed within days [89,90] are inconsistent with those obtained in months [96,97], resembling the findings in glaucoma [98,99].

In a mouse model, the expression of PKCα was decreased progressively in the cell bodies and dendrites of RBCs by the acute ocular hypertension (IOP ~100 mmHg) induced by the injection of physiological saline [100]. In the retinas injured by the intravitreal injection of 10 mM NMDA, a patch-clamp study showed that RBCs were more vulnerable to excitotoxicity than cone BCs and that PKCα critically involved the function of mGluR6. The study pointed out that in the three mice models (ocular hypertension, excitotoxic neurodegeneration, or optic nerve crash), RBC dysfunction all occurs before RGC loss. Similarly, in a glaucoma model rat, the sodium hyaluronate-induced ocular hypertension caused damage to the OPL and IPL and was associated with morphologic and morphometric changes in BCs [101]. The results indicate the vulnerability of BCs to high IOP, particularly RBCs, aligning with the high vulnerability of the peripheral/rod vision in glaucoma. It is to be further investigated whether MSCs that mediate depolarizing Na^+^ and Ca^2+^ influxes in photoreceptors and BCs can cause excitotoxicity in postsynaptic neurons, serving as an antegrade mechanism for the pressure-induced pathologies in RGCs.

PKCα speeds up light signals in RBCs [102]. Some studies have revealed that PKC can upregulate TRPV4 density and the current of TRPV4 [103,104] and TRPV1 [105] in other cells, and activation of PKCα increases the activity of TRPM1 in RBCs [106] and TRPV4 [107] in epithelial cells of pulmonary arterials. The results identify PKCα as a modulator of the pressure response of RBCs, in addition to MSCs in BCs and presynaptic neurons.

Briefly, BCs express BK, TRAAK, TREK1/2, ENaCα, and TRPV4, and glaucoma may damage BCs. It has been unclear whether other MSCs in the OPL belong to BCs. Since MSCs that are permeable to Na^+^ and Ca^2+^ may respond to pressure and mediate excitatory currents, their role in pressure-induced glutamate release from photoreceptors and BCs requires more attention and further exploration.

## 5. MSCs in ACs

In ACs from tiger salamander retinas, BK mediated miniature currents at −60 to −40 mV, which were sensitive to iberiotoxin [108]. A17 ACs from rat retinas provide reciprocal inhibitory synapses to RBCs that mediate the primary rod pathway. BK reduced GABA release from A17 ACs, regulating the flow of excitatory signals through the primary rod pathway [109]. TRPC5 is present in mouse ACs and RGCs, serving as a negative regulator of RGCs for axon outgrowth [110].

Starburst ACs (SACs) are cholinergic GABAergic ACs. They critically modulate the activities of the direction-selective RGCs and mediate the retinal wave during development. Fort and colleagues [111] observed a strong expression of the KCNK2 gene that encodes TREK1 in isolated SACs from the mouse retina. At postnatal days P1–6, TREK1^−/−^ mice exhibited an altered frequency of the retinal wave, which is known to be set by the slow afterhyperpolarization (sAHP) of SACs. The sAHP conductance was found to be calcium-dependent, reversed at the potassium reversal potential (E_K_), blocked by barium, insensitive to apamin and TEA, and activated by arachidonic acid.

Mammalian ACs express the TRAAK and TRPV2 transcripts [14,60]. The INL shows the immunoreactivity of TRAAK, TREK1, and TREK2 [52]. A low level of TRAAK is present in the IPL of salamander retina [15]. The mRNA and immunoreactivity of ENaCα were increased in the IPL and INL in D2 mice [112], and the INL of human retinas expressed the mRNA of β- and γ-subunits [113]. These studies suggest that individual interneurons require several types of MSCs to perform normal functions.

It is mostly unclear how ACs express MSCs. TRPs have been found in the IPL, including TRPV1, TRPV4, TRPV2, TRPM1, TRPM3, ENaC, and TRPC5. The IPL of mice, cats, and primates is positive for TRPV2 [114]. TRPV4 immunoreactivity is visible in the IPL of mouse [115], porcine [65], and primate retinas [31] (Table 1).

ACs have shown some changes in glaucoma retinas. Earlier studies identified the glaucoma-related thinning of OPL, loss of processes of BCs and HCs [116,117], low light sensitivity of AII ACs [118], and reduction in BC synapses in RGC dendrites [92,93,94,95] in glaucoma model mice before RGC death. GABAergic ACs, including cholinergic ACs [119], were reduced in glaucoma model D2 mice [120] and rats with elevated IOP [121]. It was concurrent with RGC death and appeared to be selective for ACs coupled to RGCs [121]. Different changes were observed in nitroxidergic (NO) ACs [120] in glaucoma models. In earlier reports, IOP elevation in rats reduced glutamate- and K^+^-induced GABA release and increased GABA uptake [122]. The data demonstrated the variation in pressure responsiveness among ACs.

In a glaucoma model rat, the sodium hyaluronate-induced ocular hypertension [101] damaged the direction-selective circuit, IPL, and OPL, as in the RGC populations. The treated eyes exhibited morphologic and morphometric changes in BCs, ON–OFF direction-selective RGCs, ON and OFF SACs, and the IPL. A recent study revealed that mobile Zn^2+^ from interneurons triggers RGC death in optic nerve injury [123], while Zn^2+^ may inhibit TRPM1 and activate TREK2 [124].

To sum up, MSCs have been observed in ACs, at least in GABAergic ones, and these MSCs can affect the function of ACs, BCs, and RGCs.

## 6. MSCs in RGCs

### 6.1. MSCs Permeable to K^+^ in RGCs

BK has been observed in RGCs from mammalian and other vertebrate retinas. It is in open states and involves the development and normal function of RGCs in vertebrates [125]. In isolated trout RGCs [125], iberiotoxin-sensitive, calcium-activated potassium currents mediated by BK were minimal before hatching but increased significantly then.

BK contributes to membrane hyperpolarization and regulates the excitability and visual signals in mammalian RGCs. In the isolated and intact RGCs [126] from ferret retinas, charybdotoxin, a blocker of BK, increased RGC spiking. Charybdotoxin and apamin (SKCa channel blocker) reduced the time to the threshold and the hyperpolarization after the spike in isolated RGCs and 80% of α- and β-RGCs. In mouse RGCs, blocking BK with charybdotoxin increased the spontaneous EPSCs and light-evoked ON-EPSCs but decreased the light-evoked OFF-IPSCs [127].

The ganglion cell layer (GCL) of the mouse retina exhibits immunoreactivities of TREK-1, TREK2 [52,128], and TRAAK [14,128]. Hughes and colleagues also observed other K2P channels in RGCs, such as TASK-1, TWIK-1, TWIK-2, and TWIK-3 [52].

In rat RGCs, TREK2 at the sites postsynaptic to GABAergic ACs may be activated by GABAB receptors to affect the activities of RGCs [129]. Using immunocytochemistry, patch-clamp, PCR, and Western blot, Zhang and colleagues [130] found that rd1 mice express higher levels of the mRNA and protein of TREK1 and TRAAK, and the arachidonic acid-evoked current was increased in RGCs, which was thought to counterbalance the depolarization of RGCs and protect the retina from excitotoxicity. TASK-3 also showed some regulation on the excitability of mouse RGCs [131]. The data suggest that K2Ps have physiological and neuroprotective roles in RGCs.

### 6.2. ENaC and Piezo in RGCs

Piezo is mechano-gated channel [21,132]. The optic nerve head expresses multiple MSCs [133]. The mRNA of Piezo1 and Piezo2 is present in the myelinated region of the mouse optic nerve, and the expression level of Piezo2 is high in the optic nerve head. Immunostaining revealed Piezo1 and Piezo2 in the GCL [134] and non-neuronal ocular tissues [134,135]. Zhu and colleagues used single-molecule fluorescence in situ hybridization (smFISH) and transgenic reporter mice expressing Piezo fusion proteins [79] to explore the distribution of Piezo in mouse retina. Piezo1 and Piezo2 were found in the GCL, trabecular meshwork, and ciliary body, and Piezo1 mRNA was more abundant. In genetically encoded Ca^2+^ indicator mice and an ex vivo pressurized retinal preparation, Harraz and colleagues reported Ca^2+^-permeable Piezo1 in the retina and cortical capillaries [135].

Piezo affects RGC functions in mouse retina. The expression of retinal Piezo2 increases in the mouse model of high IOP. Piezo1 agonist Yoda1 suppressed neurite outgrowth in RGCs. On the other hand, the Piezo antagonist GsMTx4 promoted neurite outgrowth in RGCs [134,136]. TRPC5 is a negative regulator of RGC axon outgrowth in mouse retina [110]. African descent individuals are known to experience glaucoma onset at an earlier age and develop blindness at higher rates, and a gain of function variant of Piezo1 (e756del) was revealed in 30% of African Americans compared to 0.15% for European descent. In this human cohort, the genetic variant was associated with a higher IOP level, thinning in the nerve fiber layer (NFL), and a lower optic nerve head capillary density in 1565 participants averaging 62–65 years, while the association did not reach statistical significance [137]. The peripheral retina has a lower RGC density and is more vulnerable to glaucoma. It is yet to be investigated whether Piezo1 (e756del) is associated with RGC pathologies in the peripheral retina.

The effect of MSCs is not necessarily dependent on IOP elevation. The normal- and high-tension glaucoma are diagnosable by similar pathologies at the optic disc and loss of visual field. Recent works in the congenital glaucoma model DBA/2J mice have revealed the enlarged eyeballs of RGC loss and normal IOP and normal-sized eyeballs of RGC loss and high IOP [8,138,139]. The results indicate that normal-tension glaucoma may also involve the accumulation of aqueous humor but induce retinal expansion with normal IOP. It has been clear that the increased membrane tension is a standard and suitable stimulus to open MSCs in the plasma membrane [11,140].

ENaC is a novel therapeutic target for serval human diseases [26]. It is activated by protease and blocked by amiloride [76,141]. ENaCα immunoreactivity and mRNA expression are present in the GCL and IPL in mouse retina [112] and RGCs in rat retinas [142]. GCL expresses the mRNA and protein of β- and γ-subunits of ENaC in human retinas [113]. In D2 mice, Dyka and colleagues found an upregulation of ENaCα gene expression in the IPL and GCL in D2 mouse retinas, but they did not find β- and γ-subunits [112].

### 6.3. TRPVs in RGCs

The retina expresses multiple types of TRPs of variable functional significance (reviewed by [55,56]). Gilliam and colleagues identified multiple TRPs in mouse retina with RT-PCR and immunohistology [59]. The strongest signals were reported for TRPC1, TRPC3, TRPM1, TRPM3, and TRPML1, and clear signals were obtained for TRPC4, TRPM7, TRPP2, TRPV2, and TRPV4. Other early studies have described the distribution patterns of multiple TRPs in a variety of species. Many reports are not included in this article due to the journal’s restriction.

GCL and IPL express TRPV2 in rat, cat, primate, and salamander retinas [15,31,60,143]; TRPV1 in rat and primate retinas [143,144]; and TRPV4 in mouse [115,144], porcine [65], and primate retinas [31]. The optic nerve head expressed the mRNAs of TRPV2 and TRPV4 [133]. Lakk and colleagues observed the mRNAs of TRPVs in isolated RGCs of 7–15 μm diameters from mouse retina, whose levels were TRPV4 > TRPV2 > TRPV3 and TRPV1 [145]. TRPV4 single channel conductance was measured as an inward conductance of 60 pS and outward conductance of 102 pS. TRPV3 showed a higher single-channel conductance, 172 pS at 60 mV, compared with ~100 pS for TRPV1, 2, and 4 [32].

Liedtke and colleagues first cloned cDNAs encoding the vanilloid receptor-related osmotically activated channel (VR-OAC, i.e., TRPV4) from rats, mice, humans, and chickens. They showed that TRPV4 is a cation channel gated by exposure to hypotonicity within the physiological range [67]. Later, the researchers confirmed the pressure responsiveness of TRPV4 in transfected cell lines and TRPV4 mutant mice [146,147]. Another group of scientists [30] separately reported an impaired osmotic sensation in mice lacking TRPV4.

RGCs in mouse [115] and primate retinas [31] can be activated by micromolar TRPV4 agonists GSK1016790A and 4αPDD, exhibiting membrane depolarization and higher firing rate. In cultural RGCs, TRPV4 agonists evoked calcium influxes and were associated with apoptosis of the neurons [115]. The TRPV4 antagonist RN1734 has been tested in retinal slices in culture and revealed a neuroprotective role in porcine retina [65]. These observations have confirmed the expression of TRPV4 in RGCs. The TRPV4 agonist also enhances the excitability of RGCs by shortening the delay of action potential and increasing the frequency of the excitatory postsynaptic currents. The data are consistent with the expression of TRPV4 in presynaptic BCs [31]. Li and colleagues [63] reported an increased expression of TRPV4 under high IOP. The intravitreal injection of a TRPV4 agonist induces Müller cell gliosis, and activation of TRPV4 induces the release of tumor necrosis factor-α (TNF-α) from cultured Müller cells. The inhibition of TNF-α could reduce TRPV4-mediated RGC apoptosis [63]. The results together support that RGC apoptosis involves TRPV4 located in RGCs, BCs, and Müller cells.

TRPVs also involve diabetic retinopathy. TRPV4 knockout or inhibition could prevent increased water diffusion and blood-retina barrier breakdown in the retina of streptozotocin-induced diabetic mice [148]. In rat models, the genetic deletion of TRPV2 impaired the myogenic reaction of retinal arterioles, resembling that observed in diabetic animals [149].

Sappington and colleagues reported mRNA of TRPV1 in the cell body and axon of RGCs, and the TRPV1 level increased by IOP elevation in D2 mice. They applied hydrostatic pressure (70 mmHg) to RGCs in culture and reported that TRPV1 antagonism could reduce the pressure-induced RGC apoptosis. Similar results were obtained from the RGCs of monkey, human, and rat retinas, and TRPV1 knockout and pharmacological antagonism of TRPV1 were found to prevent pressure-induced RGC apoptosis [144]. TRPV1 and TRPV4 were shown to form a protein complex in RGCs [144], but the interaction between TRPV1 and TRPV4 appears to be insignificant [145]. In isolated mouse RGCs from wild-type and *TRPV1*^−/−^ mice, Lakk and colleagues examined calcium influxes via TRPV4 by calcium imaging, and TRPV1 mutation showed no effect on TRPV4 activities evoked by the channel agonist GSK1016790 [145].

In transgenic mice where a few subtypes of RGCs were genetically labeled, the RGCs ramified in the sublaminar a showed the greatest change in the dendritic morphology after one week of IOP elevation in the microbead occlusion model of glaucoma [93]. On the other hand, our results revealed a reduced light sensitivity in both ONαGCs and OFFαGCs after a few weeks of IOP elevation [118]. Rountree and colleagues applied pulsatile injection (pulse-width > 50 ms at 0.69 kPa pressure) of Ames mediums into retinal tissue and evoked neuronal responses comparable to light responses. The response of RGCs was reduced by a TRPV blocker [150]. The genetic deletion of TRPV1 can differently affect the excitability for RGCs firing continuously to light onset (αON-Sustained) vs. light offset (αOFF-Sustained) [151]. These results support the idea that TRPVs play physiological and excitotoxic roles in RGCs.

In general, RGCs express multiple types of MSCs, and the influence of MSCs on RGC activities and survival involves not only MSCs expressed in RGCs but also MSCs in presynaptic neurons, glial cells, and blood vessels.

## 7. Other Mechanical-Sensitive TRPs in Retinal Neurons

TRPA1, TRPC1/3/5/6, TRPM3/4/7, and TRPP1 are mechano-gated channels (reviewed by [22,40]). TRPA1, TRPM1, and TRPCs mediate light response in sensory neurons. Planarians possess extraocular photoreception and display an unconventional TRPA1-mediated photophobic response to near-UV light [152]. Glutamate released from photoreceptors hyperpolarizes ON BCs by stimulating mGluR6, which was mediated by the inactivation of TRPM1 after binding with the G-protein α or βγ subunits [153,154] (reviewed by [55,155]) to mediate the light signal. TRPC6 and TRPC7 are expressed in the intrinsic photosensitive RGCs (ipRGCs) in mammalian retinas to involve melanopsin phototransduction. The melanopsin photocurrent in ipRGCs was abolished by blocking or eliminating TRPC3/6/7 channels in M1-type ipRGCs and TRPC3/6 in M2-type ipRGCs, suggesting that the target of melanopsin phototransduction varies in these ipRGCs (reviewed by [156]). TRPC1/4/5/6 are expressed in endothelial and Müller cells, and TRPC1/4/5/6(^−/−^) compound knockout mice showed resistance to diabetic retinopathy [157].

TRPC5 is present in mouse RGCs and ACs to serve as a negative regulator of RGCs for axon outgrowth [110]. The optic nerve head expresses multiple types of MSCs. The mRNA of TRPP1 and TRPP2 were observed in the myelinated region of the mouse optic nerve [133]. The optic nerve head and the glial lamina expressed TRPC1–6, TRPV2, TRPV4, TRPM1–4/6/7, TRPP1, and TRPP2. About 43% to 87% of individual astrocytes express the mRNA of TRPC1, TRPM7, TRPP1, and TRPP2. The expression level of TRPP2 was high in the optic nerve head. TRPM3 immunofluorescence was present in a subset of RGCs in mice during postnatal days 7–14 and in adult mice. The activation of TRPM3 with the synthetic TRPM3 agonist CIM0216 (CIM) induced prolonged calcium transients in RGCs, which were mostly absent in TRPM3 mutant mice. The prolonged calcium transient was not associated with strong membrane depolarizations but induced c-Fos expression [158,159].

Ischemia elicited a decrease in the ERG-record retinal responsiveness to light along with reactive gliosis and a significant increase in the expression of TRPM7 in Müller cells [160]. In another report [161], blue light triggered apoptosis of retinal pigment epithelial (RPE) cells, and its deleterious effects were partially attenuated by the synergistic action of TRPM7 and the pigment epithelium-derived factor (PEDF) via the PKC/ERK signaling pathway. The reverse transcription-polymerase chain reaction analysis demonstrated the mRNA expression of TRPC1, TRPM7, TRPV1/2/4, and TRPP1, but not TRPC6 or TRPM4. The TRPV2 inhibitor tranilast and specific TRPV2 pore-blocking antibodies reversed the hypo-osmotic stretch-induced Ca^2+^ influx in retinal vascular smooth muscle cells (VSMCs) of isolated retinal arterioles, but the inhibitors of TRPC1, TRPM7, TRPV1, and TRPV4 had no effect. The authors concluded that retinal VSMCs expressed a range of mechanosensitive TRP channels, but only TRPV2 contributed to myogenic signaling in this vascular bed [28].

## 8. Summary

Individual retinal neurons often possess several MSC types, which may mediate either depolarizing or hyperpolarizing membrane currents upon mechanical stimuli. Each MSC contributes more or less to the membrane currents or potentials, regulating visual signals or resulting in neuronal excitotoxicity. A few TRPs directly mediate phototransduction in ipRGCs and synaptic transmission in ON BCs, and some of them are also mechanosensitive in mammals. Further studies need to better characterize MSCs for their functional significance, aging change, and cooperation in physiological and pathological conditions, facilitating the development of effective therapeutic strategies for pressure-related visual disorders.

## Figures and Tables

**Table 1:** MSCs expressed in retinal layers and neurons.

MSCs	Photoreceptors	BCs, HCs, ACs, RGCs, INL, GCL	OPL, IPL	Other Cells
BK	Rods, cones, axons, synapses, salamander, goldfish, mouse, rabbit.	Cone ON BCs, RBCs, rabbit. A17 ACs, mouse. HCs, mouse, rat. RGCs, trout, ferret, mouse, salamander.	OPL, salamander, goldfish.	
TRAAK	Rods, cones, ONL, mouse.	ACs, INL, GCL, mouse.	IPL, mouse, salamander.	Müller cells, mouse.
TREK1	Photoreceptors, mouse.	INL, GCL, mouse.		
TREK2		INL, GCL, mouse.		Müller cells, mouse.
ENaC	Photoreceptors, inner and outer segments, ONL, rat, human.	GCL, rabbit, rat, human. BCs, rat, rabbit. INL, mouse, human.	IPL, OPL, rat, human, mouse.	Müller cells, rabbit. RPE, rat, human.
Piezo1/2	Piezo1, retina, guinea pig. Piezo1, rods, Xenopus.	Piezo1/2, RGCs, mouse. Piezo1, INL, mouse.		Cornea, trabecular meshwork, lens, epithelial, astrocytes, mouse. Piezo1, capillary, mouse.
TRPV2	Rods, cones, salamander. Axons, mouse.	RGCs, mouse. GCL, cat, monkey, salamander. INL (ACs), rat. HC, salamander.	OPL, mouse. IPL, OPL, rat, cat, monkey, salamander.	RPE, human, mouse, porcine. Artery, rat.
TRPV4	Rods, cones, salamander	RGCs, monkey, mouse, porcine. BCs, primate. BCs, HCs, salamander. INL(ACs), zebra fish.	OPL, IPL, monkey, mouse, porcine.	Müller cells, mouse. Microglia, mouse. RPE, human.
TRPV1	Cone and rod ribbons, goldfish, zebra fish	GCL, mouse, rat, primate. INL (ACs), rat.	OPL, mouse.	RPE, human.
TRPCs	TRPC1, rods, Xenopus	TRPC3/6/7, ipRGCs, mouse. TRPC5, ACs, mouse.	TRPC1, IPL, chicken. TRPC4, all layers, chicken.	TRPC1/4/5/6, Müller cells, endothelium, mouse.
TRPC1, TRPC3, TRPM1, TRPM3, and TRPML1, TRPC4, TRPM7, TRPP2,	Retina, mouse.	TRPM1, ON BC, mouse, human. TRPM3, GCL, mouse.	TRPM3, IPL, mouse.	TRPM7, Müller cells, rat.
TRPC1–6, TRPV2, TRPV4, TRPM1–4/6/7, TRPP1, and TRPP2				Astrocytes at optic nerve head, mouse.

Note: OPL—outer plexiform layer; IPL—inner plexiform layer; ONL—outer nuclear layer; INL—inner nuclear layer; GCL—ganglion cell layer; RPE—retinal pigment epithelium.

**Table 2:** Properties of mechano-gated channels.

	Permeability (P)	Mechanical Sensitivity	E_rev_	References
BK	Permeable to K^+^	Membrane tension	E_K_: −75 to −90 mV	[13]
TRAAK	Permeable to K^+^	Membrane tension	E_K_: −75 to −90 mV	[14,15,16,17]
TREK1, TREK2	Permeable to K^+^	Membrane stretch	E_K_: −75 to −90 mV	[16]
Piezo1, Piezo2	Permeable to monovalent and divalent cations	Membrane stretch, touch	−15 to 0 mV	[18,19,20,21,22,23]
ENaCa	PNa:PK = 100:1	Membrane stretch	−10 to 0 mV	[24,25,26]
TRPV2	PCa: PNa = 2.8	Mechanical and osmotic pressure, membrane stretch, heat > 52 °C	−3 to 10 mV	[15,27,28,29]
TRPV4	PCa: PNa = 6 to 10	Mechanical and osmotic pressure, touch, heat 27–34 °C	−10 to 0 mV	[30,31,32,33,34,35,36]
TRPV1	PCa: PNa = 10	Heat 43 to 50 °C Pressure-sensitive or -insensitive	~0 mV	[27,37,38,39]
TRPA1, TRPC1/3/5/6, TRPM3/4/7, TRPP1/2	PCa > PNa: TRPC5 (8), TRPM7 (3), TRPM3α2 (1.5) PCa < PNa: TRPA1 (0.8), TRPM4/5 (~0.01), TRPM3α1 (0.1), TRPP2 (4)	Mechanosensitive	~0 mV	[22,40,41]

## Data Availability

Not applicable.
